# Histological Hallmarks of Mucosal Healing in Inflammatory Bowel Diseases in the Era of Monoclonal Antibodies Therapy: New Insights and Perspectives

**DOI:** 10.3390/diagnostics11091570

**Published:** 2021-08-30

**Authors:** Gerardo Cazzato, Anna Colagrande, Valeria Andriola, Teresa Lettini, Sebastiano Cicco, Pragnell Mary Victoria Candance, Leonardo Resta, Leonardo Vincenti, Giuseppe Ingravallo

**Affiliations:** 1 Section of Pathology, Department of Emergency and Organ Transplantation (DETO), University of Bari “Aldo Moro”, 70124 Bari, Italy; anna.colagrande@gmail.com (A.C.); lettinit@yahoo.com (T.L.); mpragnellv@gmail.com (P.M.V.C.); leonardo.resta@uniba.it (L.R.); 2 Section of General Surgery, Department of Emergency and Organ Transplantation (DETO), University of Bari “Aldo Moro”, 70124 Bari, Italy; valeria.andriola@gmail.com (V.A.); leonardo.vincenti@policlinico.ba.it (L.V.); 3 Section of Internal Medicine, Department of Biomedical Sciences and Human Oncology, University of Bari “Aldo Moro” Medical School, Piazza G. Cesare 11, 70124 Bari, Italy; sebastiano.cicco@uniba.it

**Keywords:** IBD, biological, infliximab, adalimumab, vedolizumab, histology

## Abstract

Background: Chronic inflammatory bowel diseases (IBDs) are gaining increasing attention, both because they can severely reduce the quantity and quality of life, and because the advent of monoclonal antibodies has profoundly changed the natural history of these diseases. In recent years, the concept of mucosal healing has assumed a certain importance, and there are more and more clinical and pharmacological trials that consider this parameter among their endpoints, so much so that it may soon be included among the desirable clinical outcomes of patients with IBD. Methods: We performed a literature review of the Pubmed, Medline, and Web of Science (WoS) databases. Results: We selected 88 articles and then removed 6 duplicates; the final sample after accurate application of the inclusion criteria numbered 73 articles, with a level of evidence rating of three or four, according to Oxfords Evidence-based medicine. Our aim was to study the histological impact of monoclonal antibody therapies on mucosal healing, taking into consideration the few studies present in the literature. To perform this review, we compared studies that examined patients with Crohn’s disease (CD) and/or ulcerative colitis (UC) undergoing monoclonal therapy versus patients undergoing other non-biological therapies (PICO statements). Conclusions: We try to delineate how monoclonal antibodies have changed the natural history of IBD, acting at the microscopic level, and we believe that a careful analysis of the histopathology and the definition of the objective criteria for “Mucosa Healing” should enable this concept to be included among the clinical endpoints of patients affected by IBD, thus contributing to a better therapeutic management of these patients.

## 1. Introduction

Crohn’s disease (CD) and ulcerative colitis (UC) are the two main forms of chronic inflammatory bowel disease (IBD), an idiopathic condition characterized by a chronic fluctuating course, in which quiescent phases of variable duration are interrupted by episodes of exacerbation [1]. Although the tendency towards a different distribution of the lesions and specific clinicopathological stigmata generally allows for a differentiation between CD and UC, in about 10–15% of cases, it is not yet possible to make a clear distinction between the two conditions [1,2]. In such cases, the term “indeterminate colitis” is used: obviously, the correct diagnostic classification of IBD cannot ignore a prior exclusion of causes of non-inflammatory colitis [3,4,5]. IBDs are more frequent in industrialized countries (Northern Europe and North America), where incidence rates have begun to stabilize after a phase of progressive increase in the last 50 years [6,7]: CD is estimated at 5–6/100,000 people/year, with a prevalence of 27–105 cases/100,000, while CU is estimated at around 6–15 cases/100,000 people/year, with a prevalence of 80–150 cases/100,000 [1,6]. Conversely, incidence and prevalence rates continue to rise in low incidence areas (Southern Europe, Asia, and developing countries) [1,7].

From a microscopic standpoint, in CD erosions, fissurations and deep ulcers are observed with a transmural pattern, and the variable presence of neutrophil granulocytes, eosinophils, lymphocytes and plasma cells with a “discontinuous” pattern, as well as epithelioid and giant cell granulomas, can be seen in about 50% of cases, mainly located in the submucosa [5,6]. Instead, in UC, there is a primary involvement of the mucosa and, in the most active cases, of the submucosa. The most characteristic lesion is the formation of crypt abscesses of neutrophils which, by inducing necrosis of the crypt epithelium, generate true ulcers [7,8].

Despite the medical attention given to IBD and the considerable efforts made in researching the underlying causes, it is still not clear and fully understood what the risk factor responsible for this pathology is [1,5,6,7]. Therefore, the concept of “risk factors” linked to CD/UC, of which genetics are a part, is used, as there is evidence of, and redeeming of, genes that make a person more prone to developing Crohn’s disease than the general population. In addition, people who have a close relative (e.g., parent, brother/sister, or child) with Crohn’s disease or UC are at a higher risk of developing these conditions [6]. An abnormal reaction of the immune system to the bacteria in the intestine has been proposed, capable of creating intestinal dysbiosis and a greater chance of developing IBD. Among the environmental factors, viruses, bacteria, diet, smoking, stress, and some drugs have been studied and analyzed [1,7,8]. Furthermore, from epidemiological studies, it would seem that living in urban areas (cities) or in the more developed countries of the Northern Hemisphere, as well as belonging to the Caucasian race, may constitute an additional factor of vulnerability [1]. Finally, age plays a significant role: IBD is more likely to occur for the first time between the ages of 10 and 40, although it can begin at any age [6,7,8].

The primary therapeutic goals in a patient with CD and/or UC are to induce remission and maintenance (for as long as possible) of the quiescent state, as well as to manage the onset of any complications such as erythema nodosum, gangrenous pyoderma, migrating polyarthritis, ocular lesions, liver and biliary tract lesions, ankylosing spondylitis, or sacroiliitis [1,8,9]. The first-line inductive therapy in patients with CD/UC in the clinical mild–moderate activity phase is 5-aminosalicylic acid or Mesalazine, although molecules such as Budenoside (CD) or Prednisone and their equivalents (mostly UC) are also commonly used [10]. A valid alternative is the use of immunosuppressive drugs, such as Tioguanine derivatives (azathioprine, 6-mercaptopurine) or methotrexate and cyclosporine [8,10]. In addition to these “therapeutic weapons”, patients can now also be prescribed new biological drugs, or the so-called monoclonal antibodies, such as infliximab and adalimumab (chimeric-human and completely humanized anti-TNFalpha, respectively) or vedolimumab (anti-integrin-alfa4-beta7 expressed on the surface of a particular leukocytes subtype) [10,11]. In more detail, infliximab binds TNFalpha, an inflammatory cytokine produced by monocytes, macrophages and T lymphocytes, adhering to the membranes of Th1 lymphocytes, and is able to determine cell lysis through an antibody-dependent and/or cell-mediated toxin [10,12]. Therefore, this molecule is able to lead to the depletion of specific populations of subepithelial inflammatory cells, improving the clinical picture of IBD [12]. On the other hand, adalimumab [12,13,14,15,16,17] is able to bind with high affinity to TNF-alpha and to prevent the interaction of this inflammatory cytokine with its own receptors (p55 and p75). In addition to neutralizing the TNF-alpha present in the systemic circulation, this monoclonal antibody is able to bind the TNF-alpha expressed on the cell surface of monocytes, inducing apoptosis and lysis of these cells in the presence of complement. Finally, vedolizumab is a monoclonal anti-integrin α4β7 antibody, with selective action on intestinal lymphocyte traffic, and therefore has an innovative mechanism of action, which is based on the selective inhibition of lymphocytes that transit and are recruited into the inflamed intestine. The drug, in fact, by binding specifically to the integrin α4β7, a protein expressed in a particular subgroup of circulating white blood cells, inhibits the binding of this protein to the adhesion molecule cellular (MAdCAM-1), overexpressed in blood vessels and lymph nodes of the gastrointestinal tract inflamed. By inhibiting this bond, the drug prevents the passage of lymphocytes from the blood circulation to the intestinal wall, the site of chronic inflammation at the base of UC and MC [18,19,20,21,22,23].

In recent times, the concept of “mucosal healing” (MH) has been arising, increasing interest both because of its clinical importance and its histopathological features [9,12]. In fact, there are two types of MH: endoscopic and histological. Although there has not yet been an unambiguous definition by the authors, we tend to define endoscopic MH as “the complete absence of all inflammatory and ulcerative lesions in all segments of the intestine at endoscopy”. This definition is quite limiting [24], and does not take into due consideration the various stages of disease severity. 

Even the definition of histological MH has not yet been universally defined: the contemporary idea is to consider the absence of neutrophil granulocytes as a sign of histological mucosal healing, but there are various discrepancies in the literature regarding a precise description of this entity [18,24,25]. For these reasons, histological remission in UC and/or CD is not currently considered a clinical goal, probably also due to the complexity and/or subjectivity of application of the best known histological scores (Geboes [15], Nancy [16], Robarts), except in the research and pharmacological trials fields [12]. It is also well recognized that histological MH often does not correspond to endoscopic MH, which is why a patient can potentially still present histological IBD damage even in the absence of endoscopic signs [26]. Precisely for this reason, it is essential to acquire biopsies of the gastrointestinal tract even in the presence of endoscopic MH, as the choice of the most appropriate therapeutic treatment strictly depends on the outcome of the histological evaluation.

The advent of monoclonal antibodies (Infliximab, Adalimumab, Certolizumab, and Vedolizumab [18,23,24]) has had a strong clinical impact on primary endpoints, such as the induction and maintenance of clinical disease remission for both CD and UC [1,2,6,7,8,9,24], but also on inducing endoscopic and histological mucosal healing (MH) [24,25,26]. 

From this standpoint, the concept and definition of “mucosal healing” has a great importance in clinical practice, because recent evidence shows that MH is associated with long-term symptomatic remission and a longer relapse-free interval [27], as well as a reduction in the frequency of hospitalizations, complications and surgical resections [28], [29,30] and a significant improvement in the quality of life [31]. Additionally, MH is associated with a reduction in cancer risk and cancer-related mortality [24]. Although MH has classically been defined as the absence of ulcers “or” an improvement in endoscopic scores, such as the symptom-based Crohn’s Disease Activity Index (CDAI), Crohn’s Disease Severity Endoscopic Index (CDEIS), and/or Crohn’s Disease Simplified Endoscopic Activity Score (SES-CD) and others, all this does not faithfully depict the extent and course of the disease. Hence, an objective study of MH by histology may offer a much more adequate and effective clinical-pathological management [31,32].

In this paper, we report the main histopathological alterations in IBD described in the literature of IBD patients induced by monoclonal Ab therapy.

## 2. Materials and Methods

A systematic review was performed following the Preferred Reporting Items for Systematic Reviews and Meta-Analyses (PRISMA) guidelines. A search of PubMed, Medline, and Web of Science (WoS) databases was made for the period 2010–2021, inserting the terms Crohn’s disease and ulcerative colitis in combination with each of the following: Biological drugs; Histopathology; Mucosa healing. Only articles in English were selected. The last search was run on 26 July 2021. Eligible articles were assessed according to the Oxford Centre for Evidence-Based Medicine 2011 guidelines [20]. Review articles, meta-analyses, observational studies, letters to the editor, and comments to the letters were all included. Other potentially relevant articles were identified by manually checking the references in the included literature.

An independent extraction of the articles was performed by two investigators according to the inclusion criteria. Disagreement was resolved by discussion between the two review authors. Since the study designs, participants, treatment measures, and reported outcomes varied markedly, we focused on describing the histopathological findings, their relation to the clinical severity of the disease, use of biological drugs, and other relevant investigations. The review was performed according to the PICO statements, the characteristics of which are summarized in Table 1.

The limitations of a literature review of this nature is the complete reliance on previously published research and the availability of these studies using PRISMA guidelines.

## 3. Results

**Figure 1:**
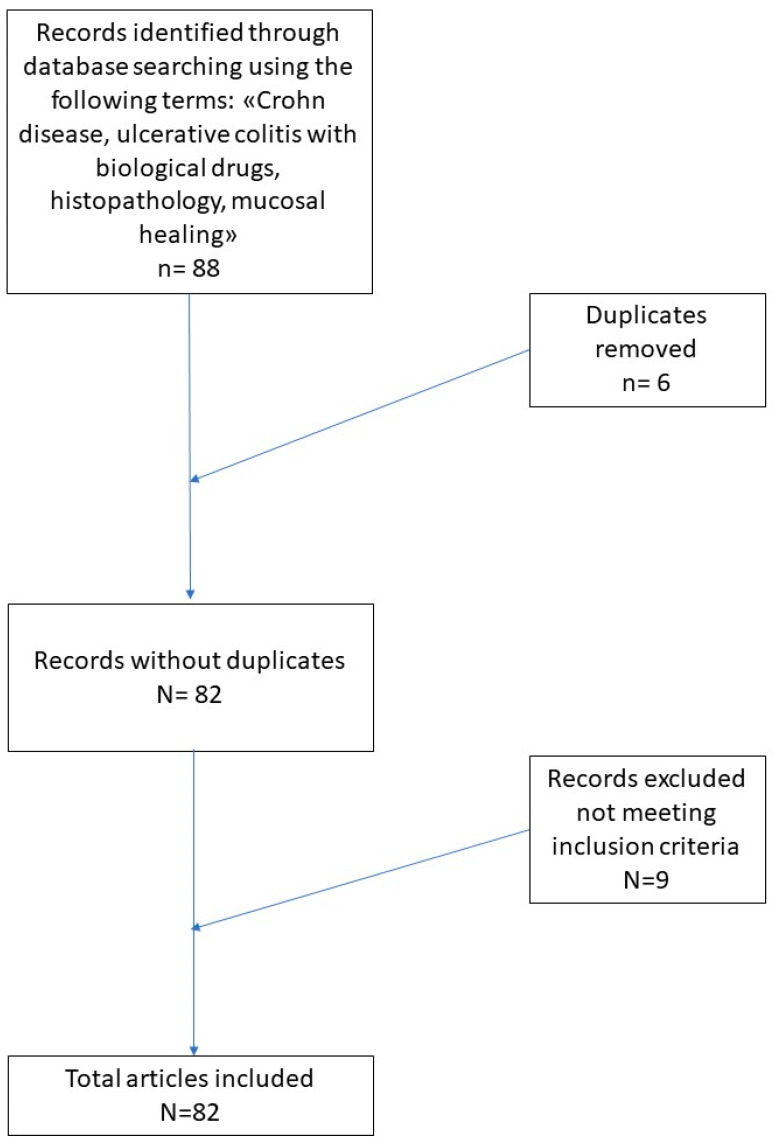
Literature search and article selection according to the PRISMA guidelines.

A total of 88 records were initially identified in the literature search, of which six were duplicates. After screening for eligibility and inclusion criteria, 73 publications were ultimately included (Figure 1). Major study and clinical characteristics are summarized in 2. The majority of publications were reviews (*n* = 44), followed by observational prospective studies (*n* = 21) and comments to letters (*n* = 8). All studies included were rated as level 4 or 5 evidence for clinical research as detailed in the Oxford Centre for Evidence-Based Medicine 2011 guidelines [20]. Table 2 summarizes the main studies used in the realization of this review.

## 4. Discussion

The histopathological definition of MH must take into account the various conditions that can influence the clinical picture: first of all, the accumulation of neutrophils in the intestinal lumen, parallel to ulcerations of the mucosa and the symptoms of IBD, with a more or less marked aggression of the glands, appear to be the “primum movens”, as they are in other districts of the gastrointestinal tract, such as the stomach [38,39,40,41,42,43,61,62,63]. Transepithelial migration of neutrophils is regulated by CD44v6 and CD55, as well as ICAM-1 (intercellular adhesion molecule-1) on epithelial cells, and has been associated with epithelial damage [44] Table 3.

Moreover, the presence of basal plasma cells has a high predictive value for the first diagnosis of IBD and is considered an important marker, especially in the differential diagnosis with other forms of colitis. As early as 1983, Scott et al. [64] demonstrated that plasma cells were increased in rectal samples from subjects with IBD compared with controls; later, Seldenrijk et al. [65] also showed that more than 50% of patients with IBD showed basal plasmacytosis compared to controls, suggesting that this parameter could be of some importance. However, successful studies have clearly shown that the inflammatory characteristics of IBD are not constant over time; for example, a prospective study showed that focal basal plasmacytosis was found in 40% of IBD patients with symptoms for <2 weeks but disappeared after 1 year of follow-up in half of those without recurrence [61]. Therefore, the “presence of plasmacells” criterion may have a dual significance, as the presence of basal plasma cells even in various phases of the disease is a sign of pre-existing IBD. In addition, eosinophils, like plasma cells, are present with variable frequencies in all phases of the disease, both in active and quiescent colitis, as recently demonstrated. For this reason, it is impossible to consider either of these cell types as an indicator of disease activity [33,34,35,36,37,45,46,64,65,66]. 

Therefore, it is correct to state that the assessment of the degree of disease activity cannot disregard the recognition, topographical description, and possible presence in the glandular (crypt abscess) of neutrophilic granulocytes, and that expressions such as “IBD in the quiescent phase” of mild/moderate disease activity may actually increase the diagnostic confusion that revolves around the concept of mucosal healing [21,23,47,48,49,50,51,52,53,54,55,56,57,58,59,60,67,68,69,70,71,72,73]. 

Despite greater attention to the concepts of endoscopic and histological healing, in most of the studies available in the literature, the assessment of the histological activity of the disease was not considered a treatment endpoint. Furthermore, the endpoints for histological remission of the disease for each patient have not been defined, therefore data on this topic are still relatively scarce.

However, D’Haens et al. [71], in a multicentre, randomized, double-blind, placebo-controlled study including 30 patients with active CD, demonstrated that patients treated with intravenous infliximab at a dose of 5, 10, or 20 mg/kg improved their endoscopic scores compared to the placebo group and, moreover, they had recieved histological healing compared to the control group, albeit with a persistence of cytoarchitectonic alterations. Baert et al. partially confirmed the ability of biologics to modify the natural history of IBD: in their study, they compared 15 patients with CD refractory to first-line therapy who were treated with intravenous infliximab with 5 placebo patients. After one month of therapy, the group of patients treated showed clear signs of histological mucosal healing, with a reduction mainly in intraglandular neutrophilic granulocytes and in the lamina propria, as well as a reduction, ascertained by immunohistochemical techniques, in CD4 +, CD8 + T lymphocytes and CD68 + macrophages [72].

Regarding the histological healing of UC patients, few studies have tried to investigate the histological changes during therapy with monoclonal antibodies [73,74]. Hassan et al. [75] studied nine patients with moderate to severe UC, treated with infliximab at a dose of 5 mg/kg, whose colon biopsies were obtained at time 0 and after 10 weeks of treatment. The activity of disease was evaluated by histological scoring, histomorphometry, and immunostaining with anti-TNFalpha antibodies. Of the nine patients studied, six responded to therapy with a marked reduction in neutrophils, crypta abscesses, inflammatory infiltration in the lamina propria, and immunostaining for TNFalpha; three non-responding patients had no histological, clinical, and endoscopic improvement. Finally, Fratila and Craciun confirmed these data, also using electron microscopy [76].

Recently Biancone et al. [77] demonstrated the results of using Ustekinumab in UC patients, establishing a secondary endpoint of histo-endoscopic mucosal healing, in which histo-endoscopic mucosal healing was defined as achieving both endoscopic improvement (Mayo endoscopic score of 0 or 1), and histological improvement (infiltration of neutrophils in <5% of the crypts, no destruction of the crypts, and no erosion, ulceration, or granulation tissue). This contribution further emphasizes the importance of histological healing in the correct evaluation of the efficacy of monoclonal Ab.

Regarding new biological drugs, in a recent paper by Arijs et al. [18], the authors have shown that Vedolizumab (VDZ) induces histological healing in >50% of patients with endoscopic healing, with a maximum effect at week 52. VDZ also restored, although incompletely, the colonic expression of many immune-related genes in UC patients who achieved endoscopic healing at week 52. However, persistent histological and genetic dysregulations remained even in healing patients, suggesting that maintenance therapy will be needed to control intestinal inflammation. On other hand, other new molecules are increasingly entering into initial clinical use, as reported by Schmitt et al. [68]. Cobitolimod may be a new therapeutic approach in UC, as it suppresses Th17 cells and induces anti-inflammatory IL10 + macrophages and regulatory T cells, thus modifying the balance of dysregulated intestinal cytokines through an agonist-type action relative to Toll-like receptor 9. Table 4.

From the analysis of all the papers examined for this review, it seems correct to state that it is only relatively recently that controlled and randomized studies relating to histopathological modifications from monoclonal antibody therapy have begun to be carried out. In particular, it would seem that there are “common” effects on mucosal healing with regard to Infliximab, Adalimumab, and Vedolizumab, both in CD and in UC (reduction of inflammatory infiltrate in the chorion, restoration of the mucus-secreting capacity of the colon glands, restoration of the functions of the intestinal barrier), but also more specific effects of the molecule in question (for example, Vedolizumab, which acts selectively on B and T lymphocytes, downregulating their mucosal involvement). Moreover, from the review of the literature, it emerges clearly that anti-TNFalpha monoclonal antibodies induce a histological healing of the mucous membranes superior to previously developed therapies through two main mechanisms of action represented by the induction of apoptosis of T cells in the chorion and going to “reprogram” the effector functions of monocytes/macrophages in the direction of the M2 line, making them able to mediate a real mucosal histological healing [78].

## 5. Conclusions

A correct methodological approach to the evaluation of colon biopsies, in addition to the availability of comprehensive clinical and endoscopic data, are essential. In this sense, an adequate and correctly oriented number of biopsies is of fundamental importance, as highlighted in an ECCO ESP 1 statement: “For a reliable diagnosis of inflammatory bowel disease, it is necessary to perform ileo-colonoscopy rather than rectoscopy. At least two biopsies to be performed. at least five sites along the colon, including the rectum, and the terminal ileum should be performed, biopsies which should be, possibly oriented correctly, on cellulose acetate filters: ECCO Statements 4A and 4B.”.

Histologically, the presence or absence of neutrophils must be considered as the distinctive sign of differentiation between the active and the quiescent phase of the disease, and an expression of the efficacy of therapy (histological healing of the mucosa).

To reach a greater inter-observer agreement among different pathologists, it is necessary to avoid any form of morphological score in the evaluation of the colon mucosa, because, as has been amply demonstrated, these are currently all extremely complicated and subjective.

We therefore believe that, in the near future, “histological mucosal healing” should be considered as a target for therapy in IBD and as an important remission endpoint to be achieved, together with clinical, laboratory, and endoscopic signs of improvement.

## Figures and Tables

**Table 1:** PICO statements utilized in this review.

PICO Facets	Considerations
Patient (P)	Persons with histological diagnosis of IBD (CD e/o UC)
Intervention (I)	Therapy with monoclonal antibody
Comparison (C)	Therapy without monoclonal antibody
Outcome (O)	Histological difference in remission induced by Ab-monoclonal therapy compared to other therapies

**Table 2:** Main studies used in the realization of this review.

Number of Reference	Author(s)	Year(s)	Type of Paper	Therapeutic Treatment	Endpoint of MH (Y/N)	Modification of MH
[1]	Baumgart et al.	2007	Review	Not applicable	Not applicable	Not applicable
[2]	Sairenji et al.	2017	Review	Not applicable	Not applicable	Not applicable
[9]	Boal Carvalho et al.	2017	Review	Not applicable	Not applicable	Not applicable
[18]	Arijs et al.	2018	Clinical Trial	Vedolizumab	Yes	55% responders
[19]	Dai et al.	2014	Clinical Trial	Infliximab	No	Not applicable
[28]	Fiorino et al.	2011	Review	Not applicable	Not applicable	Not applicable
[33]	Neurath et al.	2012	Review	Not applicable	No	Not applicable
[34]	Ferrante et al.	2012	Review	Monoclonal Ab	Yes	Yes
[35]	Rogler et al.	2012	Review	Monoclonal Ab	Yes	Yes
[36]	Seidelin et al.	2013	Review	Monoclonal Ab	Yes	Yes
[37]	Osterman	2013	Review	Various therapy	Yes	Not applicable
[38]	Dulai et al.	2015	Review	Monoclonal Ab	No, but desirable	Not applicable
[39]	Florholmen	2015	Review	Monoclonal Ab	Yes	Yes
[40]	Yu et al.	2015	Original article	Infliximab (only CD)	Not, but desirable	Not applicable
[41]	Shah et al.	2016	Review with meta-analysis	Infliximab, Adalimumab Vedolizumab	Yes	Yes
[42]	Vickers et al.	2016	Review	Monoclonal Ab (only UC)	Yes	Not applicable
[43]	Reinink et al.	2016	Review	Various therapies	No	Not applicable
[44]	Eder et al.	2016	Original article	Monoclonal Ab	Yes	Yes
[45]	Cholapranee et al.	2017	Review	Monoclonal Ab	Yes	Yes
[46]	Kurashima et al.	2017	Review	Various therapies	Not applicable	Not applicable
[47]	Pantavou et al.	2019	Meta-analysis	Monoclonal Ab and Tofacinib (only UC)	Yes	Yes
[48]	Singh et al.	2018	Review	Monoclonal Ab	Yes	Yes
[49]	Leppkes et al.	2018	Editorial	Various therapies	No	Not applicable
[50]	Antonelli et al.	2018	Review	Monoclonal Ab and others oral therapies	Yes	Not applicable
[51]	Castiglione et al.	2019	Original article	Monoclonal Ab (only CD)	No	Not applicable
[52]	Park et al.	2019	Original article	Monoclonal Ab	No	Not applicable
[53]	Samaan et al.	2019	Review	Monoclonal Ab	Yes, deep histological remission	Yes
[21]	Pigneur et al.	2019	Randomized controlled trial	Monoclonal Ab (only childrens with CD)	Yes	Yes
[54]	Löwenberg et al.	2019	Original article	Vedolizumab (only CD)	Yes	Yes, 64% of patients
[22]	Li K et al.	2019	Clinical trial	Ustekinumab (only CD)	Yes	Yes
[55]	Pouillon et al.	2019	Review	Vedolizumab (only UC)	Yes	Yes
[56]	Cucchiara et al.	2020	Review	Monoclonal Ab	Yes	Not applicable
[23]	Nardone et al.	2020	Review	Monoclonal Ab	Yes	Not applicable
[57]	Petryszyn et al.	2020	Review	Infliximab Adalimumab Vedolizumab Tofacitinib (only UC)	Yes	Yes
[58]	Kucharzik et al.	2020	Review	Monoclonal Ab	Not applicable	Not applicable
[59]	Sagami et al.	2020	Comparative study	Monoclonal Ab	Yes	Not applicable
[60]	Li et al.	2020	Review	Ustekinumab (only UC)	Yes	Not applicable

**Table 3:** Main features of CD versus UC. It should be noted that, in about 25–30% of cases, it is not easy to discriminate between the two diseases.

	Clinical Findings	Histological Findings
Crohn disease (CD)	Perianal lesion common; frank bleeding less frequent than in UC	Transmural discontinous inflammation with fissuring, submucosal involvement, granuloma (25–28%), pseudopiloric metaplasia, globet cells preservation
Ulcerative colitis (UC)	Bloody diarrhea	Acute and chronic diffuse inflammatory infiltrate, depletion of globet cells, crypt abscesses, lymphoid aggregates, distorsion of crypts, basal plasmacytosis

**Table 4:** Main histological features in MH described in monoclonal antibodies therapy in IBD.

Crohn Disease	Clinical MH Features	Histological MH Features
Infliximab	Reduction of Crohn Disease index of severity (CDEIS) and simple endoscopic score for Crohn disease (SES-CD)	Reduction of inflammatory infiltrate at normal levels. Reduction of epithelial damage. Persistence of crypt architecture.
Adalimumab	Reduction of Crohn’s disease activity index (PCDAI) and simple endoscopic score for Crohn disease (SES-CD)	Reduction of inflammatory infiltrate at normal levels. Reduction of epithelial damage.
Vedolizumab	Reduction of Crohn Di-sease index of severity (CDEIS) and simple endoscopic score for Crohn disease (SES-CD)	Reduction of inflammatory infiltrate with reduction of neutrophils. Reduction of epithelial damage. Persistence of crypt architecture.
Ustekinumab	Reduction of simple endoscopic score for Crohn disease (SES-CD)	Reduction of global histology activity scores (GHASs).
Ulcerative Colitis		
Infliximab	Reduction of Mayo Endoscopic Score (MES)	Reduction of alterations of the intestinal epithelium, such as depletion of microvilli, crushing of epithelial junctions, cytoplasmic vacuolization. Restoration of the function of intracellular organelles. Reduction of pycnotic nuclei. Restoration of muciparous goblet cells with regular mucus formation and secretion.
Adalimumab Golimumab	Reduction of Ulcerative Colitis Endoscopic Index of Severity (UCEIS)	Restricting the inflammatory infiltrate and T-cell proliferation within the lamina propria. Downregulation of the expression of metalloproteinases and proinflammatory molecules. Restore the protective capabilities of the mucosa by reinforcing intestinal permeability and mucosal secretion, activating fibroblasts, and maintaining epithelial regeneration.
Vedolizumab	Ulcerative Colitis Endoscopic Index of Severity (UCEIS)	Limits both B- and T-cell lymphocyte fixation on the intestinal vascular endothelial cells and consequent migration to the lamina propria and tissue cells.

## Data Availability

Not applicable.
